# Supplementary material for: Does Androgen Deprivation for Prostate Cancer Affect Normal Adaptation to Resistance Exercise?
Source: Int J Environ Res Public Health. 2022 Mar 23;19(7):3820. doi: 10.3390/ijerph19073820 (PMC8997930; doi:10.3390/ijerph19073820)
Supplement: Supplementary file 1 [file ijerph-19-03820-s001.zip › ijerph-1635992-supplementary.pdf]

International Journal of
Environmental Research and Public Health

MDPI

Supplement: Risk of bias evaluation

Allocation

Random sequence generation

All studies in prostate cancer (PCa) patients reported enough information to be evaluated as low risk of bias regarding randomization sequence generation [1–7]. Contrary, for studies in healthy elderly men (HEM), where limited details for the randomization processes were available, which precluded the risk of bias judgments. Seven studies stated that participants were randomized without describing the process in more detail [8–14] two studies provided sufficient information to be judged as having a low risk of bias in randomization sequence generation [15,16].

Allocation concealment

All studies in PCa cancer patients reported enough information to be evaluated as low risk of bias regarding allocation concealment [1–7]. Five studies in HEM provided insufficient information to evaluate the risk of bias in allocation concealment [8–14] and two studies provided sufficient information to be judged as having a low risk of bias [15,16].

Blinding

Blinding of participants and staff

Due to the nature of exercise, blinding participants is not possible; therefore, all studies in PCa patients and HEM were judged to have a high risk of bias in blinding participants and study staff.

Blinding of outcome assessments

Although participants could be instructed not to disclose group allocation during an exercise test (e.g., the one-repetition maximum test), the fact that participants allocated to intervention would be familiarized with the test equipment could preclude proper blinding of instructors. However, studies using independent test personnel were still judged as having a low risk of bias. Only objectively measured endpoints were included in the present meta-analysis.

Among the seven studies in PCa patients, five studies did not provide information on blinding of outcome assessments [1–3,6,7]. Nilsen et al. (2015) [4] and both studies from Winters-Stone et al. (2015; 2016) [5,6] both report using blinded personnel for DXA scans, but Nilsen et al. (2015) [8–16] did not report using blinded personnel for assessing muscle strength. For studies in HEM, only Solberg et al. (2013) [15] reported sufficient information on blinding of outcome assessments to be judged as having a low risk of bias. Since the same personnel was used as training instructors and to supervise assessments of muscle strength Deibert et al. (2010) [12] was judged to a have a high risk of bias. The remaining studies did not report sufficient information to allow for the risk of bias judgments8-[10,13,14,16].

Incomplete data reporting

Studies reporting attrition rates of >20% were judged to have a high risk of bias in reporting incomplete data.

For studies in PCa patients, most studies were judged to have a low risk of bias regarding incomplete data [1–3,5,6]. However, Nilsen et al. (2015) [4] reported DXA and muscle strength assessments of only 75% of participants included in the intervention group. Furthermore, Newton et al. (2019) reports 26% and 30% drop-out from the intervention group (that is relevant for this meta-analysis) and from the delayed aerobic group (control group), respectively.

For studies in HEM, Ades et al. (1996) [8] and Lovell et al. (2010) [11] did not report specifically on recruitment and attrition rates, but no drop-outs were reported from these studies. Solberg et al (2013) [15] reported high attrition rates from a "functional strength training group", but this group is not included in the present meta-analysis. Finally, the remaining studies reported sufficient information to be judged as having a low risk of bias regarding incomplete data [9,10,12–14,16].

Selective reporting

Due to the narrow scope of the present meta-analysis, our inclusion- and exclusion criteria yielded a highly selected cohort of studies. Therefore, all of the included studies in both PCa patients [1–7] and HEM [8–16] were judged to have a low risk of bias regarding selective reporting of variables relevant for this meta-analysis.

Other biases

Potential sources for contamination of intervention effects often relate to adherence to the allocated study group, both for the intervention groups and the control groups. Reporting of adherence to resistance training, or exercise training in general, is often limited to attendance rates, and consequently, no other option is available for the risk of bias judgments. Importantly, studies reporting similar amounts of physical activity between exercise- and control groups will not be judged as having a high risk of other biases in the present meta-analysis. The rationale behind this is the specificity of the effect of resistance exercise on changes in lean body mass and muscular strength.

Three studies in PCa patients were judged to have a low risk of bias [1,3,4]. It should, however, be noted that during the discussion section Nilsen et al. (2015) [4] reported no change in exercise behaviour in the control group, but this statement is not supported with data. Cormie et al. (2015) [2] described a home component of the exercise prescription but did not report adherence to this part of the intervention, and the risk of bias was therefore judged as unclear. Winters-Stone et al. (2015) [5] reported low attendance (<80%) to the home component of the exercise intervention but sufficient attendance to the supervised component (>80%). Although it is unclear whether this would have an impact on LBM changes, it precludes proper judgment of the risk of bias. Finally, Winters-Stone et al. (2016) [6] reported attendance rates <80%.

For studies in HEM, Ades et al. (1996) did not report adherence/attendance to the exercise intervention [8]. The duration of the planned 26-week intervention period was extended by four weeks in 11 of 51 participants in the study by Stewart et al. (2005) [9]. This could indicate compromised feasibility of the planned intervention but would most likely not introduce any risk of bias. The remaining studies reported sufficient information to be judged as having a low risk of other biases [10–16].

Supplementary Reference

1. Alberga AS, Segal RJ, Reid RD, et al: Age and androgen-deprivation therapy on exercise outcomes in men with prostate cancer. Support Care Cancer 20:971-81, 2012
2. Cormie P, Galvao DA, Spry N, et al: Can supervised exercise prevent treatment toxicity in patients with prostate cancer initiating androgen-deprivation therapy: a randomised controlled trial. BJU Int 115:256-66, 2015
3. Galvao DA, Taaffe DR, Spry N, et al: Combined resistance and aerobic exercise program reverses muscle loss in men undergoing androgen suppression therapy for prostate cancer without bone metastases: a randomized controlled trial. J Clin Oncol 28:340-7, 2010
4. Nilsen TS, Raastad T, Skovlund E, et al: Effects of strength training on body composition, physical functioning, and quality of life in prostate cancer patients during androgen deprivation therapy. Acta Oncol:1-9, 2015
5. Winters-Stone KM, Dieckmann N, Maddalozzo GF, et al: Resistance Exercise Reduces Body Fat and Insulin During Androgen-Deprivation Therapy for Prostate Cancer. Oncol Nurs Forum 42:348-56, 2015
6. Winters-Stone KM, Lyons KS, Dobek J, et al: Benefits of partnered strength training for prostate cancer survivors and spouses: results from a randomized controlled trial of the Exercising Together project. J Cancer Surviv 10:633-44, 2016
7. Newton RU, Galvao DA, Spry N, et al: Exercise Mode Specificity for Preserving Spine and Hip Bone Mineral Density in Prostate Cancer Patients. Med Sci Sports Exerc 51:607-614, 2019
8. Ades PA, Ballor DL, Ashikaga T, et al: Weight training improves walking endurance in healthy elderly persons. Ann Intern Med 124:568-72, 1996
9. Stewart KJ, Bacher AC, Turner KL, et al: Effect of exercise on blood pressure in older persons: a randomized controlled trial. Arch Intern Med 165:756-62, 2005
10. Kukuljan S, Nowson CA, Sanders K, et al: Effects of resistance exercise and fortified milk on skeletal muscle mass, muscle size, and functional performance in middle-aged and older men: an 18-mo randomized controlled trial. J Appl Physiol (1985) 107:1864-73, 2009
11. Lovell DI, Cuneo R, Gass GC: The effect of strength training and short-term detraining on maximum force and the rate of force development of older men. Eur J Appl Physiol 109:429-35, 2010
12. Deibert P, Solleder F, Konig D, et al: Soy protein based supplementation supports metabolic effects of resistance training in previously untrained middle aged males. Aging Male 14:273-9, 2011
13. Timmons JF, Minnock D, Hone M, et al: Comparison of time-matched aerobic, resistance, or concurrent exercise training in older adults. Scand J Med Sci Sports 28:2272-2283, 2018
14. Ziegler AK, Jensen SM, Schjerling P, et al: The effect of resistance exercise upon age-related systemic and local skeletal muscle inflammation. Exp Gerontol 121:19-32, 2019
15. Solberg PA, Kvamme NH, Raastad T, et al: Effects of different types of exercise on muscle mass, strength, function and well-being in elderly. Eur J Sports Sci:112-125, 2013
16. Tieland M, Verdijk LB, de Groot LC, et al: Handgrip strength does not represent an appropriate measure to evaluate changes in muscle strength during an exercise intervention program in frail older people. Int J Sport Nutr Exerc Metab 25:27-36, 2015.
